# Inside the genome: understanding genetic influences on oxidative stress

**DOI:** 10.3389/fgene.2024.1397352

**Published:** 2024-06-25

**Authors:** Hari Krishnan Krishnamurthy, Imbaasree Rajavelu, Michelle Pereira, Vasanth Jayaraman, Karthik Krishna, Tianhao Wang, Kang Bei, John J. Rajasekaran

**Affiliations:** ^1^ Vibrant Sciences LLC., San Carlos, CA, United States; ^2^ Vibrant America LLC., San Carlos, CA, United States

**Keywords:** oxidative stress, reactive oxygen species, genetic polymorphisms, prooxidants, antioxidants, repair genes

## Abstract

Genetics is a key factor that governs the susceptibility to oxidative stress. In the body, oxidative burden is regulated by the balance between the prooxidant genes that orchestrate processes that produce oxidant species, while the antioxidant genes aid those involved in scavenging these species. Together, the two components aid in maintaining the oxidative balance in the body. Genetic variations can influence the expression and activity of the encoded proteins which can then affect their efficiency in regulating redox processes, thereby increasing the risk of oxidative stress. This review studies single nucleotide polymorphisms (SNPs) that bear relevance to oxidative stress by exploring the variations in the prooxidant genes, such as XDH, CYBA, CYP1A1, PTGS2, NOS, and MAO and antioxidant genes including SOD, CAT, GPX, GSS, GLUL, GSR, GSTM1, GSTM5, GSTP1, TXN and HMOX1. Early identification of individuals at the increased risk of oxidative stress is possible from the assessment of sequence of these genes. Integrating genetic insights into oxidative stress management measures can pave the way for personalized medicine that tailors’ healthcare approaches to individual genetic profiles. Effective genetic assessment along with routine quantification of biological markers can improve and monitor treatment strategies, enhancing mitigation approaches that maintain cellular health and promote longevity.

## Introduction

Oxygen is a fundamental element for life and plays a crucial role in extracting energy through oxidation processes in the human body. This metabolic necessity, while essential, concurrently gives rise to transient entities, including reactive oxygen species (ROS) and reactive nitrogen species (RNS), primarily originating from the mitochondria (Scandalios, 2005). Although pivotal for immune defense and cellular signaling, an excess of ROS and RNS can harm the body by modifying lipids, DNA, RNA, and proteins, instigating detrimental oxidative reactions (Scandalios, 2005). To counteract oxidative damage, the human body has evolved a sophisticated antioxidant defense mechanism comprising endogenous and exogenous antioxidants. Antioxidants play a crucial role in protecting against oxidative stress by preventing the formation of reactive species, scavenging, neutralizing, and removing reactive species, inhibiting oxidative chain reactions, and chelating reactive metals, therefore combatting oxidative stress. (Scandalios, 2005). Oxidative stress (OS) is defined as an imbalance between oxidants and antioxidants in favor of the oxidants, leading to a disruption of redox signaling and molecular damage (Scandalios, 2005).

Oxidative stress has garnered attention due to its association with the onset and/or progression of several diseases, including cancer, diabetes, metabolic disorders, atherosclerosis, and cardiovascular diseases (CVD) (Bhattacharyya et al., 2014). Elevated levels of ROS and RNS attack cellular macromolecules which can trigger fundamental changes at the cellular level, leading to chronic inflammation, DNA damage, and disruptions in cell signaling pathways. Alterations in these critical pathways can be associated with the pathogenesis of various diseases (Ramírez-Expósito and Martínez-Martos, 2019). For instance, in cardiovascular diseases like atherosclerosis, oxidative stress can contribute to the development of arterial plaques (Scandalios, 2005). In diabetes, oxidative stress is implicated in insulin resistance and pancreatic beta-cell dysfunction (Scandalios, 2005). Persistent oxidative stress can also contribute to the development and promotion of cancer by causing genetic mutations, promoting angiogenesis, and facilitating metastasis (Andreadi et al., 2022). The global prevalence of these disorders underscores the significance of screening for oxidative stress.

Oxidative stress is known to be influenced by intrinsic elements such as genetic predispositions and epigenetic modifications (Barry, 2018). Prooxidant genes such as *XDH, CYBA, CYP1A1, PTGS2, NOS, MAO* encoding the enzymes, xanthine oxidase, NADPH oxidase, CYP1A1 enzyme, and cyclooxygenase-2, nitric oxide synthase, and monoamine oxidase, respectively, are involved in the generation of reactive species. Variations in these genes might pose the risk of higher oxidant production. Antioxidant genes, such as *SOD, CAT,* and *GPX* encode to the primary antioxidant enzymes, superoxide dismutase, catalase, and glutathione peroxidase, respectively. Various enzymes involved in the glutathione system encoded by genes, *GSS, GLUL, GSTM1, GSTM5, GSTP1* as well as the thioredoxins (*TXN*) and heme oxygenase–1 (*HMOX1*) contribute to the body’s antioxidant system by maintaining redox homeostasis. Polymorphisms in these antioxidant genes can affect their activity and efficiency, thereby affecting the body’s antioxidant defences. For instance, variations in the *SOD* gene influence the enzyme’s function and reduce its activity, increasing the susceptibility to oxidative stress (Gusti et al., 2021).

Understanding the genetics of prooxidants and antioxidants can provide insights into the genetic factors influencing oxidative stress-related disease risk. Genetic assessment for these genes might enable the early identification of individuals at higher risk of oxidative stress. Moreover, the exploration into the genetic landscape opens a promising avenue for personalized medicine, where interventions can be tailored based on an individual’s unique genetic profile.

### Overview of oxidative stress - oxidants and antioxidants

Prooxidant substances encompass both endogenous and exogenous compounds that instigate oxidative stress either by generating ROS or by impeding the function of antioxidant defense mechanisms (Di Meo and Venditti, 2020). Within the delicate equilibrium of cellular redox homeostasis, prooxidant genes play a significant role, often tipping the scale towards oxidative stress under certain conditions (Checa and Aran, 2020; Di Meo and Venditti, 2020). Among these genes, XDH, MAOB, NOS, and the enzymatic machinery involving NADPH and CYP1A1 stand out as key contributors to the generation and propagation of ROS (Checa and Aran, 2020).

To counteract oxidative stress, the body employs mechanisms involving enzymatic and non-enzymatic antioxidants. Antioxidants are encoded by genes that scavenge or eliminate a variety of free radicals including those generated during biological processes, thereby maintaining redox balance (Huchzermeyer et al., 2022). Among these defenders, *SOD, CAT,* and *GPX* genes serve as frontline protectors, neutralizing ROS and preventing their deleterious effects. Additionally, *GSS, GLUL, GSR,* and Glutathione S-transferases (*GSTM1, GSTM5, GSTP1*) collaborate to replenish and utilize glutathione, a vital antioxidant molecule, in the detoxification of ROS and xenobiotics. Furthermore, *TXN* and *HMOX1* contribute to the antioxidant defense network by regulating redox-sensitive signaling pathways and degrading heme, respectively (Shaw and Chattopadhyay, 2020).

### Navigating oxidative stress: signalling pathways and their mediators

In response to oxidative stress, various signaling pathways orchestrate a complex cellular defense mechanism aimed at restoring the balance between oxidants and antioxidants. One prominent pathway is the Nrf2/ARE (nuclear factor erythroid 2-related factor 2/antioxidant response element) pathway (Liang et al., 2017; Liu et al., 2022). Under normal conditions, Nrf2 is sequestered in the cytoplasm by Keap1 (Kelch-like ECH associated protein 1). However, upon exposure to oxidative stress, Nrf2 dissociates from Keap1 and translocates into the nucleus, where it forms complexes with other transcription factors such as c-Jun and small Maf proteins. These complexes bind to the ARE in gene promoters, triggering the expression of over 200 genes involved in cellular defense against oxidative stress and inflammation. These genes include antioxidative enzymes like HO-1, which play a pivotal role in restoring the redox state balance by scavenging reactive oxygen species (ROS) (Liu et al., 2022).

Additionally, the NF-κB signaling pathway is activated in response to oxidative stress, regulating cellular proliferation and apoptosis in inflammatory states. NF-κB can induce the expression of specific genes that attenuate ROS production and promote cell survival. For instance (Liang et al., 2017; Liu et al., 2022), NF-κB activation may lead to the upregulation of genes encoding antioxidative enzymes such as SOD2 and GPX4 (Liu et al., 2022). Furthermore, the PI3K/AKT pathway modulates vascular tone by regulating the production of nitric oxide (NO) through the phosphorylation of endothelial nitric oxide synthase (eNOS). Dysregulation of this pathway under oxidative stress conditions can result in endothelial cell injury, highlighting the intricate interplay between oxidative stress and vascular function (Liu et al., 2022).

Another set of signaling pathways involved in oxidative stress response includes ferroptotic, apoptotic, FoxO, and ErbB pathways (Liang et al., 2017; Liu et al., 2022). These pathways collectively regulate cellular responses to oxidative stress, with key players such as FoxO3a and p53 orchestrating antioxidant responses by promoting the expression of antioxidative enzymes like SOD2 and GPX1 (Liu et al., 2022). Moreover, ErbB receptors, particularly EGFR and ErbB2, are implicated in the induction of oxidative stress and subsequent cellular survival mechanisms. Activation of these receptors can lead to the transcriptional activation of Nrf2, further enhancing the expression of antioxidant and detoxification proteins. Overall, these signaling pathways act in concert to mount a robust antioxidant response against oxidative stress, thereby safeguarding cellular integrity and function (Liang et al., 2017; Liu et al., 2022).

### Prooxidant genes in oxidative stress

The generation of oxidant species involves both enzymatic and nonenzymatic reactions (Hajam et al., 2022). Enzymatic reactions in the respiratory chain, prostaglandin synthesis, phagocytosis, and the cytochrome P450 system significantly contribute to ROS production. Key enzymes, including NADPH oxidase, and xanthine oxidase play crucial roles in synthesizing superoxide radicals (O_2_^⋅-^), leading to the formation of hydrogen peroxide (H_2_O_2_), hydroxyl radicals (^⋅^OH), peroxynitrite (ONOO^−^), and hypochlorous acid (HOCl). H_2_O_2_ is a nonradical compound, generated by various oxidase enzymes, such as amino acid oxidase and xanthine oxidase. The highly reactive hydroxyl radical (^⋅-^OH) is formed through the interaction of O_2_^⋅-^ with H_2_O_2_, catalysed by Fe^2+^ or Cu^+^ in the Fenton reaction. Additionally, the nitric oxide radical (NO^⋅^) is enzymatically synthesized from the oxidation of arginine to citrulline by nitric oxide synthase (NOS) (Hajam et al., 2022). Nonenzymatic reactions also contribute to free radical production, especially during mitochondrial respiration, where oxygen reacts with organic compounds. Exposure to toxins and ionizing radiation trigger nonenzymatic free radical formation (Hajam et al., 2022).

In this review, we delve into the molecular mechanisms underlying the involvement of *XDH, MAOB, COX-2 NOS, NADPH,* and *CYP1A1* in oxidative stress, exploring their roles in cellular redox balance and their implications in various disease states.

### Xanthine oxidase

**FIGURE 1:**
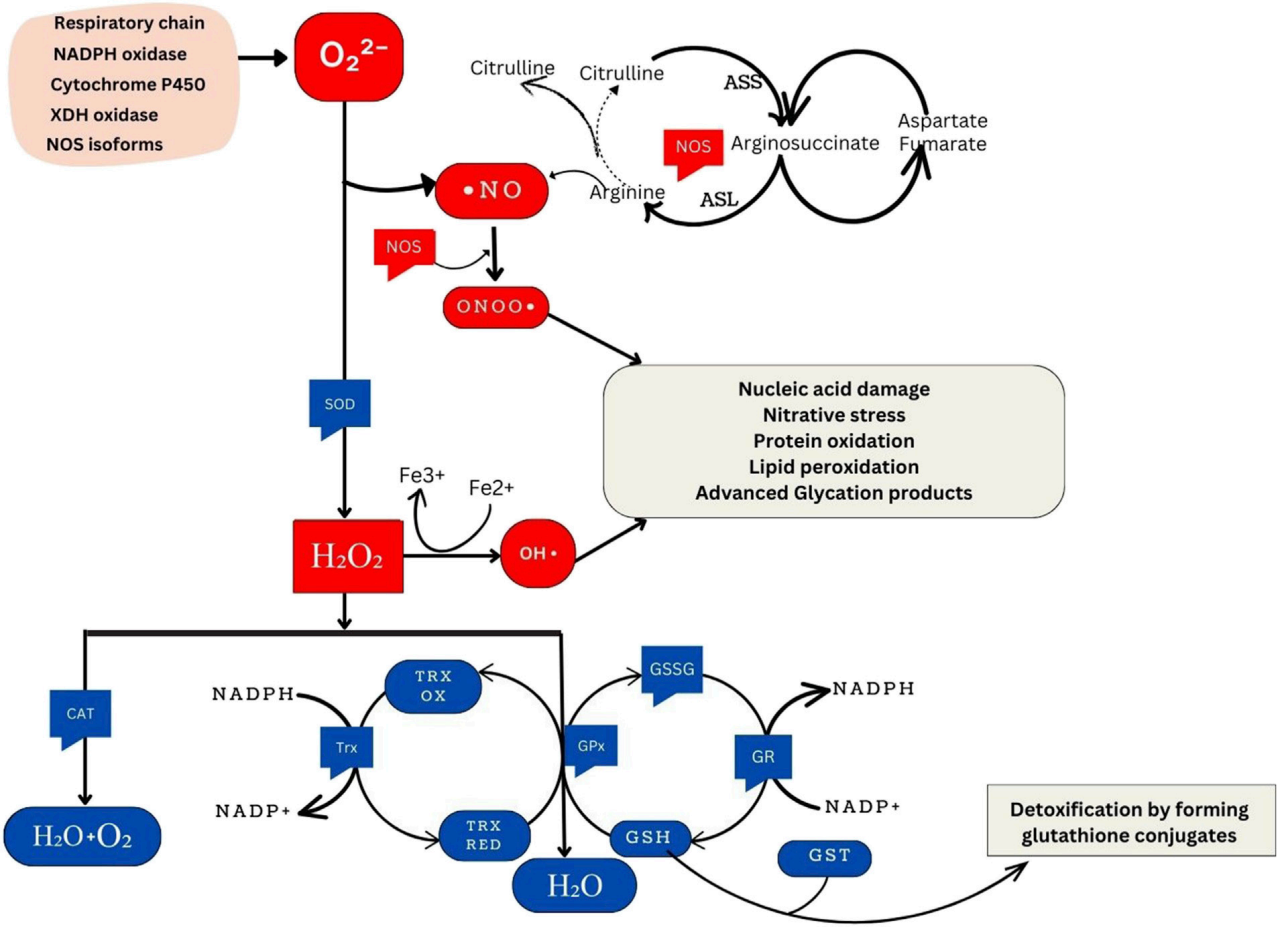
Overview of Cellular Oxidative Stress Pathways and Antioxidant Defense Mechanisms. NADPH oxidase, an essential component of the immune response, generates superoxide. Similarly, xanthine oxidase produces superoxide during purine metabolism. The respiratory chain, integral to cellular energy production, generates superoxide as a byproduct of electron transport. Cytochrome P450 enzymes, involved in metabolic processes, also contribute to superoxide production. Under certain conditions, nitric oxide synthase isoforms can produce superoxide, particularly when uncoupled. ⋅NO is synthesized from the citrulline cycle by NOS enzymes. These enzymes catalyze the conversion of L-arginine to L-citrulline and NO, a process involving the oxidation of L-arginine’s guanidino nitrogen. When NO reacts with O_2_^⋅-^, ONOO− forms, a highly reactive nitrogen species implicated in oxidative damage to biomolecules. SOD enzymes convert superoxide radicals into H_2_O_2_. These include cytosolic Cu/Zn-SOD (SOD1), mitochondrial Mn-SOD (SOD2), and extracellular SOD (SOD3). Hydrogen peroxide can produce ⋅OH through the Fenton reaction, a process catalyzed by transition metal ions such as Fe^2+^. This reaction initiates chain reactions leading to oxidative damage. Catalase (CAT), located in peroxisomes, breaks down hydrogen peroxide into water and oxygen. This catalysis facilitates the conversion of hydrogen peroxide molecules into water and oxygen molecules. Moreover, the glutathione and thioredoxin systems directly reduce H_2_O_2_ to water. In the glutathione system, glutathione peroxidase (GPx) reduces H_2_O_2_ using reduced glutathione (GSH) as a cofactor. Similarly, thioredoxin peroxidase (TPx) reduces hydrogen peroxide using thioredoxin as a cofactor. Both systems protect cells from oxidative damage by detoxifying hydrogen peroxide. GSH and glutathione S-transferase (GST) are crucial in detoxifying harmful compounds. Through conjugation, GSH binds to electrophilic centers on toxic molecules, enhancing their water solubility and facilitating excretion from cells. This detoxification process aids in protecting cells from the damaging effects of xenobiotics and reactive oxygen species (ROS).

Xanthine oxidase (XO) is a molybdoflavoprotein hydroxylase that can act both as an oxidase (XO) and reductase (called xanthine dehydrogenase). It is encoded by the *XDH* gene. Both forms of the enzyme aid in the final stage of purine catabolism. They catalyze the last two oxidative reactions that convert hypoxanthine to xanthine and xanthine to uric acid, a well-known antioxidant. However, this process results in the generation of O_2_^⋅-^ and H_2_O_2_ (Kumar et al., 2018) (Figure 1). Additionally, XO is involved in the hydroxylation of various substrates and the production of NO^⋅^ under hypoxic conditions from nitrates and nitrites (Harrison, 2002). This increases the availability NO^⋅^ to react with O_2_^⋅-^ to give ONOO^−^ radicals (Harrison, 2002). This dual functionality of XO, in participating in the synthesis of uric acid and in being a source of ROS, underscores its significance in oxidative stress pathways.

$$\text{Hypoxanthine}+H_{2}O+O_{2}\;⇌\text{ Xanthine}+H_{2}O_{2}$$

$$\text{Xanthine}+H_{2}O+O_{2}\;⇌\text{ Uric acid}+H_{2}O_{2}$$

Polymorphisms in the prooxidant, *XDH* gene associated with its increased activity results in higher ROS and RNS production leading to oxidative stress. Increased production of ROS by *XDH* has been described in experimental models of salt-sensitive, and glucocorticoid-induced hypertension (Laakso et al., 1998). Some studies have suggested that *XDH* activity is enhanced in patients with hypertension and a higher production of H_2_O_2_ mediated by XDH in hypertensives as compared with controls has been described (Suzuki et al., 1998). Among several *XDH* polymorphisms, variants at positions 565 + 64 CT and −337 GA are of particular interest. Individuals carrying specific genotypes, such as the CC genotype of the 565 + 64 CT polymorphism, have been found to exhibit higher levels of oxidative stress markers, including malondialdehyde (MDA) and eight-oxo-deoxyguanosine (8-oxo-dG) as compared to individuals with the CT and TT genotypes. This suggests that C allele enhances oxidase function and may predispose individuals to increased oxidative stress, potentially contributing to a range of oxidative stress-related conditions (Alderman et al., 1999). Similarly, the −337 GA polymorphism has shown associations with oxidative stress markers, primarily through elevated MDA levels seen among individuals with AA and AG genotypes in comparison with the GG genotypes. These studies strengthen the potential role of *XDH* variants in the risk of oxidative stress and related diseases such as hypertension (Chaves et al., 2007).

### NADPH oxidase

NADPH oxidase (NOX) is a transmembrane enzyme located in intracellular organelles. NOX is a transmembrane enzyme and is involved in the production of O_2_^⋅-^ during cellular processes. However, exposure to diverse stimuli can amplify the production of ROS from these processes which leads to oxidative stress. The p22phox subunit, originating from the *CYBA* gene, plays a vital role in NOX function by stabilizing the catalytic subunit and providing a docking site for cytosolic factors, thereby facilitating NADPH oxidase activity (Aviello and Knaus, 2018). Upon translocation to the membrane and co-localization with p22phox and other NADPH subunits (p67phox, p47phox, and p40phox), NADPH oxidase stands out as the sole known enzyme family dedicated to producing ROS as its primary function. NOX orchestrates the transfer of electrons from cytosolic NADPH, traversing through FAD to penetrate the membrane *via* hemes, reaching oxygen and resulting in O_2_^⋅-^ generation in the cytoplasm. Therefore, the *CYBA* gene through NADPH oxidase is involved in maintaining cellular processes by the generation of ROS (Kumar et al., 2015) (Figure 1).

$$\text{NADPH}+2O_{2}\;{\text{NADP}}^{+}+2\;O_{2}^{•-}+H^{+}$$

Numerous genetic polymorphisms have been reported within the promoter and exonic regions of the *CYBA* gene. Some of these polymorphisms influence gene expression and subsequently, NADPH oxidase activity, leading to elevated free radical formation. Among several *CYBA* polymorphisms, rs4673 (C242T), rs9932581 (A-930G), and rs8854 variants have been extensively studied (Ortega-Loubon et al., 2021). In the promoter region, G alleles of rs9932581 and T alleles of rs8854 are associated with increased promoter activity, resulting in elevated oxidative stress (Tupurani et al., 2018). These polymorphisms are in the potential binding site of C/EBP (CCAAT/enhancer-binding protein) transcription factors, suggesting their role in modulating *CYBA* promoter activity and influencing *CYBA* transcription (San José et al., 2008). Studies have linked these genetic variations to susceptibility to oxidative stress-related diseases like hypertension, accompanied with increased oxidative stress markers such as eight-isoprostaglandin F_2_α (8-isoPGF2α) levels, along with reduced antioxidant CAT activity (Tupurani et al., 2018). For the rs4673 polymorphism, different rates of O_2_^⋅-^ production have been demonstrated depending on the genotype. The T allele is associated with reduced NADPH oxidase activity, both at basal levels and when stimulated. This allele has been suggested to confer protection against oxidative stress pathologies (Najafi et al., 2012). Studies in patients with obstructive sleep apnea indicate that the CC genotype associates with higher oxidative marker levels, such as 8-Isoprostane levels, while the TT genotype associates with lower eight-isoPGF2α levels. This suggests that individuals with the CC genotype exhibit higher *CYBA* activity and experience increased oxidative stress compared to those with TT genotypes (Kheirandish-Gozal and Gozal, 2013). These genetic variations exert a significant influence on oxidative stress markers, antioxidant activity, and disease susceptibility.

Another single nucleotide polymorphism (SNP), rs3017887, located in the 5′untranslated region (UTR) of the Nox4 gene, has been associated with alanine aminotransferase (ALT) levels, a marker of inflammatory activity. Notably, the NOX4 CA and AA genotype of rs3017887 exhibited a significant association with ALT levels. Furthermore, a statistically significant difference in genotype frequencies was observed when the population was stratified by steatosis and nonalcoholic steatohepatitis (NASH) (Rabelo et al., 2018). The presence of the A allele of rs3017887 has been implicated in disease susceptibility, likely attributed to increased necrotic activity in the context of heightened oxidative stress. Elevated ALT levels are indicative of hepatocyte damage in nonalcoholic fatty liver disease (NAFLD). Importantly, oxidative stress, although more pronounced in cirrhotic patients, is not solely a late-stage phenomenon but is presumed to occur early, even when transaminase levels remain elevated. These findings suggest that functional polymorphisms influencing inflammation and/or oxidative stress may serve as heritable markers associated with other allelic variants predisposing individuals to oxidative stress and inflammation (Rabelo et al., 2018).

### Cytochrome P450 family 1 subfamily a member 1

Cytochrome P450 (CYP) enzymes, specifically the CYP1A subfamily constitute a diverse group that plays pivotal roles in metabolizing both, internally generated (endobiotic) and foreign (xenobiotic) substances within the human body (Danielson, 2002). *CYP1A1* belongs to this subfamily and it is mainly in extrahepatic tissues where it participates in the metabolism of a vast number of endobiotics and xenobiotic such as toxins and drugs. However, *CYP1A1*’s metabolic activity also results in the generation of ROS as byproducts, particularly when metabolizing certain procarcinogens like polycyclic aromatic hydrocarbons (PAHs) found in environmental pollutants and food contaminants. The overexpression of *CYP1A1* usually caused due to exposure to PAHs results in the increase in ROS generation (Figure 1). CYP1A1 has been shown to regulate intracellular iron levels and contribute to the production of ROS through the Fenton reaction. As a result, variations in this gene might have implications in oxidative stress (Stading et al., 2020).

$$\text{Fenton Reaction}:{\text{Fe}}^{3+}+H_{2}O\;\to \;{\text{Fe}}^{2+}+{\text{OH}}^{•}+H^{+}$$

The *CYP1A1* gene exhibits the polymorphism, rs4646903, located in the 3′-UTR. In rs4646903, the T>C alteration influences the enzyme’s activity which results in the increase in *CYP1A1* activity (Kiyohara et al., 2012). As a result, individuals with variant genotypes (CC and TC) may experience higher ROS production during metabolic reactions as opposed to those with the wild-type genotype (TT) having optimum *CYP1A1* activity (Kiyohara et al., 2012). It is fair to infer that the rs4646903 polymorphism predisposes the CC and TC genotypes to increased oxidative stress. This inference was supported by the observed increase in the levels of the oxidative marker, MDA and the decrease in the antioxidant, GPx (Peddireddy et al., 2016). The SNP can predispose these individuals to diseases associated with oxidative damage, such as chronic obstructive pulmonary disease (COPD) and coronary artery disease (Kim et al., 2018).

### Cyclooxygenase-2

The *PTGS2 or COX-2* gene which is responsible for encoding the cyclooxygenase-2 enzyme, plays a crucial role in susceptibility to oxidative stress. The enzyme contributes to the production of inflammatory molecules by catalysing the conversion of arachidonic acid into prostaglandins, specifically, prostaglandin G2 and prostaglandin H2. This process results in the generation of O_2_^⋅-^ and subsequently other oxidant species. Moreover, *COX-2* expression is upregulated during oxidative stress and inflammation. This creates a positive feedback loop where *COX-2* activity is further boosted increasing the production of pro-inflammatory prostaglandins, which exacerbate oxidative stress and tissue damage (Simmons et al., 2004; Jaén et al., 2018). Variations in the *COX-2* gene can be implicated in oxidative stress-related conditions such as cancer, cardiovascular diseases, and neurodegenerative disorders.

Polymorphisms within the *COX-2* gene, such as rs20417 (−765G > C) significantly influence oxidative stress (Kim et al., 2016). The rs20417 polymorphism situated upstream from the transcription start site of the *COX-2* gene introduces a critical alteration in a stimulatory protein binding site. This genetic variation leads to a consequential increase in transcription activity, resulting in elevated expression of the *COX-2* enzyme (Benelli et al., 2018). The heightened expression of *COX-2*, in turn, is known to play a significant role in the intricate relationship between oxidative stress and cancer susceptibility (Benelli et al., 2018). By converting arachidonic acid into prostaglandins, *COX-2* becomes a key player in oxidative stress-mediated inflammation and cytokine production. The CC genotype of rs20417 is associated with a higher incidence of oxidative stress (Roberts et al., 2009; Katkam et al., 2019). This polymorphism has been linked to a higher risk of colorectal and gastric diseases, potentially due to increase in oxidative stress levels (Rudnicka et al., 2019; Gholamalizadeh et al., 2022).

### Nitric oxide synthase

The family of nitric oxide synthase (NOS) proteins, which includes neuronal NOS (*nNOS or NOS 1*), inducible NOS (*iNOS or NOS 2*), and endothelial NOS (*eNOS or NOS 3*), plays a crucial role in catalyzing the oxidation of L-arginine, producing ^⋅^NO and L-citrulline (Supplementary Figure S1). These enzymes, encoded by separate genes, significantly contribute to cellular redox balance and various cellular functions (Cinel et al., 2002). ^⋅^NO, a multifaceted molecule, acts as a chain-breaking antioxidant in free radical-mediated lipid peroxidation. Optimal levels of ^⋅^NO are important for vasodilation, host defence, and other cellular signaling processes in the body. Generally, concentrations ranging from pico to nanomolar levels are considered the optimum range for ^⋅^NO, where it positively influences various physiological processes. It is crucial to know that the oxidative status of the underlying tissue can affect ^⋅^NO synthesis and bioavailability. When endogenous tissue oxidant levels are high (particularly, O_2_^⋅-^) they attack ^⋅^NO to form the cytotoxic, ONOO^−^ (Supplementary Figure S1). This reduces ^⋅^NO levels aggravating NO-dependent oxidative stress. However, studies have proposed ^⋅^NO to represent a ‘double-edged sword’ with its overproduction leading to a multitude of ^⋅^NO by-products implicated in mutational events and carcinogenesis. It is hypothesized that metabolic oxygen and nitrogen species from ^⋅^NO may attack DNA bases, resulting in point mutations, strand breaks and interactions with sulfhydryl groups potentially leading to carcinogenesis (Chaaben et al., 2015). This indicates the need for maintaining ^⋅^NO at optimal levels. Therefore, understanding the role of NOS proteins is essential as their genetic variations can impact ^⋅^NO production.

N•O+O2•ˉ → ONOO−

Polymorphisms in the different *NOS* genes have been studied for their association with oxidative stress. Located in the intron region of the *NOS1* gene, the C allele of the rs1879417 (g.117803515C > T) polymorphism was associated altered *NOS1* function leading to increased oxidative stress. This allele correlated with an increased risk of oxidative stress-related conditions, such as stroke, when compared to individuals with T alleles (Synowiec et al., 2021). In the *NOS2* gene, three SNPs in the promoter region, namely, −1659 C>T (rs8078340), −1026G>T (rs2779249), and −277A>G (rs2779248) contribute to increase ^⋅^NO production (Chaaben et al., 2015). Specifically, the T alleles of rs8078340 and rs2779249, along with G alleles of rs2779248, lead to higher ^⋅^NO production. These “high ^⋅^NO expressor” variants raise ^⋅^NO levels, potentially resulting in the generation of ROS and contributing to oxidative stress. Elevated concentrations of ^⋅-^NO under certain circumstances can generate ONOO^−^ which is toxic and has carcinogenic potential (Chaaben et al., 2015). These polymorphisms are associated with conditions such as hypertension, diabetes mellitus, stroke, hypercholesterolemia, atherosclerosis, cardiovascular diseases, and kidney diseases. Polymorphisms in the *NOS3* genes such as T-786C (Pacanowski et al., 2009), G894T (Glu298Asp) (Kondkar et al., 2020), and 27bp-VNTR (Nath et al., 2009) are linked to altered ^⋅^NO production leading to oxidative stress. For the SNPs, T-786C and G894T, the homozygous (*NOS3*−786 CC) and/or heterozygous (*NOS3* 894GT + TT) states are significantly associated with the with low ^⋅^NO and high oxidative stress (Chaaben et al., 2015). Similarly, 27bp-VNTR is seen to result in low ^⋅^NO bioavailability leading to disease progression (Chaaben et al., 2015). These polymorphisms, identified as low NO expressor alleles/genotypes, result in a global reduction in ^⋅^NO production due to a 50% reduction in promoter activity. This reduction in ^⋅^NO levels contribute to the observed heightened oxidative stress in individuals carrying these risk alleles/genotypes.

### Monoamine oxidase - B

Monoamine oxidases (MAOs) are mitochondrial enzymes that oxidize monoamines, producing H_2_O_2_ and reactive aldehydes. There are two isoforms: MAO-A and MAO-B, with MAO-B playing a key role in regulating intracellular redox balance. Disruptions in monoamine metabolism and genetic variations in the *MAO* genes can cause oxidative stress, affecting cellular redox balance (Kaludercic et al., 2014; Ramsay, 2016). Among the *MAO-B* gene polymorphisms, rs1799836 is of great importance. This polymorphism is in intron 13 of the *MAO-B* gene and is thought to disrupt monoamine metabolism, leading to increased ROS production and oxidative stress within the central nervous system (Leko et al., 2021). In this polymorphism, the enzymatic activity of MAO-B is affected; the A allele is associated with elevated MAO-B activity, while the G allele is linked to lower MAO-B activity. Studies consistently show that individuals with the AA genotype exhibit higher MAO-B enzyme activity and protein levels, confirming the involvement of the A allele in heightened oxidative stress through increased MAO-B expression (Leko et al., 2021). The implications of rs1799836 extends to various neurodegenerative diseases such as Parkinson’s Disease (PD) and mental health conditions like bipolar disorder and panic disorder, mediated by the A allele’s effect in oxidative stress (Angelopoulou et al., 2022). Table 1 provides a gist of the mechanisms by which genetic polymorphisms influence prooxidant genes.

**TABLE 1:** Mechanisms of genetic polymorphisms affecting prooxidant genes.

Prooxidant enzyme	Gene name	rsID	Function of mutation	References
Xanthine Oxidase	XDH	565 + 64CT, −337GA	Enhanced XDH activity, shifting towards oxidase function, and disrupting redox balance	Kumar et al. (2015), Aviello and Knaus (2018)
NADPH oxidase	CYBA	rs9932581, rs8854, rs4673	Increased CYBA promoter activity leading to enhanced NADPH oxidase function giving rise to high O_2_^⋅-^ levels	Danielson (2002), Najafi et al. (2012), Kheirandish-Gozal and Gozal (2013), Rabelo et al. (2018)
Cytochrome P450 enzymes	CYP1A1	rs4646903	Increased CYP1A1 activity resulting in higher ROS production	Peddireddy et al. (2016), Kim et al. (2018)
Cyclooxygenase-2	PTGS2	rs20417	Altered protein binding site and increased transcriptional activity result in the elevated expression of COX-2 enzyme leading to increased ROS production	Roberts et al. (2009), Benelli et al. (2018), Katkam et al. (2019), Gholamalizadeh et al. (2022)
Nitric oxide synthase	NOS1	rs1879417	Altered NOS1 function leading to increased oxidative stress	Kondkar et al. (2020)
	NOS2	rs8078340, rs2779249, rs2779248	Altered NOS2 activity leading to higher ^⋅^NO production implicated in oxidative stress	Synowiec et al. (2021)
	NOS3	T-786C, G894T, 27bp-VNTR	Altered NOS3 activity leading to lower ^⋅^NO production implicated in oxidative stress	Nath et al. (2009), Kaludercic et al. (2014), Ramsay (2016), Synowiec et al. (2021)
Monoamine oxidases	MAO-B	rs1799836	Disrupted monoamine metabolism associated with elevated MAO-B activity leading to increased ROS production	Reuter et al. (2010)

Abbreviations: O_2_^⋅-^, superoxide; ROS, Reactive oxygen species.

^⋅^NO, Nitric oxide; NOS1 - Nitric oxide synthase–1; NOS2 - Nitric oxide synthase–2; NOS3 - Nitric oxide synthase–3; MAO-B, Monoamine oxidase - B.

### Antioxidant genes in oxidative stress

Antioxidants neutralize excess free radicals, protecting cells and contributing to disease prevention (Reuter et al., 2010). Endogenous antioxidants are classified as enzymatic antioxidants and non-enzymatic antioxidants. The primary antioxidant enzymes, including superoxide dismutase (SOD), catalase (CAT), and glutathione peroxidase (GPx) directly neutralize ROS and RNS. SOD catalyzes the dismutation of O_2_^⋅-^ into H_2_O_2,_ which is then transformed into water (H_2_O) and oxygen (O_2_) by CAT or GPx. Glutathione is an integral antioxidant in the body and it orchestrates its antioxidant functions with the help of various enzymes that together form the ‘glutathione system.’ Glutamate-cysteine ligase (GLUL) catalyzes the formation of the precursor to GSH while glutathione synthetase (GSS) is one of the enzymes participating GSH synthesis. GPx removes H_2_O_2_ by using it to oxidize reduced glutathione (GSH) into oxidized glutathione (GSSG) (Reuter et al., 2010) (Figure 1).

Glutathione reductase (GR) regenerates GSH from GSSG utilizing NADPH as a source of reducing power (Andreadi et al., 2022). The glutathione enzyme family, glutathione S-transferases (GSTs) also contribute to glutathione-mediated antioxidant actions. Additionally, the thioredoxin system comprising thioredoxin (Trx) and thioredoxin reductase (TR) mediate antioxidant functions by using NADPH. Heme oxygenase (HO) is another important enzyme that regulates oxidative stress by maintaining heme homeostasis (Figure 1). In addition to these internal enzymatic antioxidant defences, the body also has non-enzymatic antioxidants that are further divided into endogenous non-enzymatic antioxidants (e.g., glutathione, alpha-lipoic acid, coenzyme Q10, melatonin, uric acid, bilirubin) and exogenous non-enzymatic antioxidants (e.g., vitamin A, E, C, selenium, zinc, carotenoids, trace metals, flavonoids, omega-3 and omega-6 fatty acids) (Andreadi et al., 2022).

The enzymatic and non-enzymatic antioxidants together mount effective antioxidant defences against oxidant species in the body. The antioxidant process operates through chain-breaking or prevention mechanisms. In chain-breaking, antioxidants stabilize free radicals formed during reactions, preventing further damage, while in prevention, antioxidant enzymes reduce the rate of chain initiation, scavenging initiating free radicals or stabilizing transition metal radicals (Scandalios, 2005). This intricate process is critical for maintaining redox homeostasis and preventing oxidative damage (Scandalios, 2005).

This review explores the multifaceted roles of the enzymatic antioxidant genes in mitigating oxidative stress, unravelling their intricate mechanisms of action and their significance in maintaining cellular health and resilience.

### Superoxide dismutase

Superoxide dismutase (SOD) is a group of enzymes found in oxygen-dependent organisms that convert the highly reactive, O_2_^⋅-^ into less reactive, H_2_O_2_ and oxygen (O_2_) through redox reactions of metal ions within their active sites (Barry, 2018) (Figure 1). This is the integral mechanism by which SOD reduces oxidative stress in the body. Humans have three distinct SOD isoforms: copper-zinc superoxide dismutase (Cu/ZnSOD) or SOD1, manganese superoxide dismutase (MnSOD) or SOD2, and extracellular superoxide dismutase (ECSOD) or SOD3 (Ściskalska et al., 2020). Higher levels of SOD can enhance the antioxidant defense system, reducing oxidative damage to cells and potentially lowering the risk of various diseases, including cancer and neurodegenerative disorders such as Alzheimer’s disease (Aranda-Rivera et al., 2022; Saxena et al., 2022).

$$O_{2}^{•-}+O_{2}^{•-}+2H_{2}O\overset{\mathrm{Cu/ZnSOD}}{\to }H_{2}O_{2}+O_{2}$$

The *SOD1* gene encodes for the enzyme, superoxide dismutase one present in cellular compartments (Eleutherio et al., 2021). Polymorphisms in the *SOD1* gene have garnered attention due to their impact on oxidative stress regulation. One extensively studied polymorphism is rs2234694 (+35A/C), situated at the junction site between the intron and exon 3 (Ben Anes et al., 2019; Gallegos-Arreola et al., 2020). The AA genotype is associated with an increase in SOD1 enzyme activity, while the CC genotype correlates with a reduction in enzymatic activity. This reduction in enzyme activity can lead to a compromised ability to catalyze the conversion of O_2_^⋅-^ into H_2_O_2_ and O_2._ As a result, the balance in the ROS levels is disrupted, contributing to an increased susceptibility to oxidative stress (Gallegos-Arreola et al., 2020). Another notable polymorphism, rs36232792 is the 50 bp Insertion/Deletion (Ins/Del) located 1,684 base pairs upstream of the ATG start codon in the *SOD1* gene promoter region. The Del allele in this polymorphism is linked to a reduction in promoter activity which can result in decreased synthesis of the SOD1 enzyme, compromising the its ability to neutralize O_2_^⋅-^ radicals (Mirsadraee and Saadat, 2019; Gallegos-Arreola et al., 2020; Sedaghat et al., 2024). This reduction in enzymatic activity and compromised ROS detoxification might contribute to an elevated oxidative stress environment within the cell (Mirsadraee and Saadat, 2019). The implications of these *SOD1* gene polymorphisms extend to various diseases such as heart failure, cancer, diabetes, Down’s syndrome, and amyotrophic lateral sclerosis owing to their roles in altered redox signaling.

*SOD2* encodes superoxide dismutase two that neutralizes O_2_^⋅-^ generated during oxidative phosphorylation (Flynn and Melov, 2013). The *SOD2* polymorphism, rs4880 located in exon 2, introduces a T to C substitution at position 2,734, resulting in the SOD2 Ala16Val genotype. The Val allele, a product of this SNP, significantly reduces SOD2 activity within the mitochondria *via* the accelerated degradation of SOD2 Val mRNA. As a result, individuals with the Val variant may experience higher oxidative stress. On the other hand, the Ala variants are associated with higher SOD2 Ala mRNA synthesis in cells, thereby having optimum antioxidant function. Additionally, the mitochondrial targeting sequence (MTS) of the SOD2 Ala precursor facilitates efficient mitochondrial import through an α-helix conformation while the MTS of the SOD2 Val precursor, adopting a β-sheet structure, results in a less efficient transport. Consequently, SOD2 activity is approximately 40% higher following the mitochondrial import in the SOD2 Ala precursor compared to its Val counterpart (Pourvali et al., 2016). The *SOD2* rs4880 polymorphism is believed to be associated with the susceptibility to various diseases, including cancer, neurodegenerative disorders, chronic kidney disease (CKD), and cardiovascular diseases (Gao et al., 2008; Pourvali et al., 2016; Griess et al., 2017).

The *SOD3* gene encodes for superoxide dismutase three playing a pivotal role as an extracellular antioxidant enzyme. Particularly abundant in the lungs, it contributes significantly to SOD activity in the airways and blood vessels protecting lung tissues from oxidative stress. (Gongora et al., 2008). The *SOD3* gene, particularly in exon 3, is linked to a commonly studied SNP, specifically rs1799895 (R213G polymorphism). This SNP occurs in the heparin-binding domain of the *SOD3* gene, leading to an arginine-to-glycine amino acid substitution at position 213 (R213G). The genetic variations among CC genotypes resulting from this polymorphism witness an impaired binding of ECSOD to the extracellular matrix, leading to lower tissue levels of the enzyme in comparison to individuals carrying CG and GG genotypes (Sørheim et al., 2010). This reduction in ECSOD levels results in decreased protection of lung matrix components against oxidative damage, indicating a potential involvement in the progression of chronic obstructive pulmonary disease (COPD) and a decline in lung function over time (Gongora et al., 2008; Sørheim et al., 2010).

### Catalase

The *CAT* gene encodes the catalase enzyme, primarily found in cell peroxisomes and the cytoplasm. It plays a crucial role in breaking down H_2_O_2_ produced during cellular respiration into oxygen and water (Figure 1). Catalase is consistently active in systems involved in electron transport with cytochromes, where H_2_O_2_ formation poses a threat to cellular integrity (Ighodaro and Akinloye, 2018). Genetic variations within the *CAT* gene, particularly in its promoter region and coding sequence, can affect catalase activity and may influence an individual’s susceptibility to oxidative stress-related diseases (Kodydková et al., 2014).

$$2H_{2}O_{2}\overset{\text{CAT}}{\to }2H_{2}O+O_{2}$$

Variations in the *CAT* gene, including, −262C>T (rs1001179) (McCullough et al., 2012), −844C/T or −844G/A (rs769214) (Wu et al., 2023), and C111T (rs769217) (Hebert-Schuster et al., 2012) polymorphisms have been widely studied. These polymorphisms, located in the promoter region are associated with alterations in catalase expression levels and activity. Specifically, the rs1001179 polymorphism has been linked to variations in catalase levels and activity affecting the enzyme’s ability to neutralize intracellular H_2_O_2_. Carriers of the TT-genotype of the *CAT* gene rs1001179 polymorphism exhibited lower levels of catalase activity compared to carriers of CT- and CC-genotypes, suggesting a potential role in oxidative stress (McCullough et al., 2012). The other polymorphism, rs769214 has been associated with higher CAT activity in basal conditions, depending on the binding site of the transcriptional factor PAX6. The T allele of this polymorphism has been linked to increased CAT transcriptional activity (Wu et al., 2023). The rs769217 is responsible for alterations in CAT activity, with individuals carrying the TT genotype associated with lower CAT activity compared to those with the wild-type allele (Hebert-Schuster et al., 2012). While the variant allele in rs769214 is improving the enzyme’s activity, the variant allele of rs769217 is reducing CAT activity reading to oxidative stress.

### Glutathione peroxidase

Glutathione Peroxidase (GPx) catalyzes the reduction of H_2_O_2_ to water and oxygen (Figure 1). It also reduces peroxide radicals (ROO^⋅^) to alcohols and oxygen. Inactivity of GPx can result in oxidative damage and trigger inflammatory pathways associated with nuclear factor-κB (NF-κB) (Shaparov et al., 2021). GPx comprises at least eight different members in humans, labeled GPx1 to GPx8 (Flohé and Brigelius-Flohé, 2011). Most GPx enzymes use selenocysteine as a cofactor. While not all of them have selenocysteine, they all rely on GSH in their active sites. GPx5, GPx7, and GPx8 lack selenocysteine and instead use cysteine (CysGPxs). They are called thioredoxin-dependent peroxidases and use cysteine (Cys) in their redox-active sites (Flohé and Brigelius-Flohé, 2011). Due to their integral role in antioxidant activity, polymorphisms in GPx are implicated in various conditions, including cancer, hypertension, vitiligo, neurodegenerative diseases, and cardiovascular disease. (Toppo et al., 2008).

$$H_{2}O_{2}+2\text{GSH}\overset{\text{GPx}}{\to }2H_{2}O+\text{GSSG}$$

$$\text{ROO}•+2\text{GSH}\overset{\text{GPx}}{\to }\text{ROH}+H_{2}O+\text{GSSG}$$

Glutathione Peroxidase 1 (GPx1), also known as cellular GPx, is encoded by the *GPX1* gene and plays a crucial role in antioxidant defence mechanisms (Lubos et al., 2011; Mohammedi et al., 2016). A notable polymorphism in the *GPX1* gene, rs1050450, is a leucine to proline change at codon position 198 (GPX1 Pro198Leu genotype). This SNP involves a C>T substitution at position 198, resulting in the replacement of proline (Pro) with leucine (Leu). The presence of the Leu allele in the *GPX1* gene can affect the protein’s catalytic enzyme activity, substrate affinity, and structural stability. Specifically, the GPX1 Leu variant exhibits lower enzymatic activity compared to the GPX1 Pro enzymes which may weaken its ability to combat oxidative stress (Lubos et al., 2011; Mohammedi et al., 2016; Jerotić, 2021).

Glutathione Peroxidase 3 (GPx3) encoded by the *GPX3* gene, is primarily released into the extracellular space. GPx3 serves as a crucial antioxidant enzyme in the vasculature. Its main function involves maintaining a delicate balance between various oxidant species and ^⋅^NO, a key vasorelaxant maintaining endothelial health (Chang et al., 2020). Therefore, GPx3’s role is essential in establishing an antithrombotic vascular environment, averting endothelial dysfunction, and reducing the likelihood of diseases associated with oxidative stress. Studies have identified the *GPX3* gene to be associated with the risk of arterial ischemic stroke, cerebral venous thrombosis, and sudden sensorineural hearing loss (SSNHL), potentially due to its genetic influence on ROS (Voetsch et al., 2008; Chien et al., 2017). For the rs3805435 in the *GPX3* gene, individuals with the AA genotypes exhibited a deficiency in the GPx3 enzyme, leading to heightened extracellular oxidant stress, platelet activation, poor antioxidant defenses, and potential oxidative modification of fibrinogen compared to the AG and GG genotypes (Voetsch et al., 2008; Chien et al., 2017). This sequence of events increases the risk of oxidative stress-related diseases, including acute ischemic stroke, hypertension, platelet-dependent thrombosis, coronary artery disease, and SSNHL (Voetsch et al., 2008; Chien et al., 2017).

Glutathione Peroxidase 4 (GPx4) encoded by the *GPX4* gene, is a crucial antioxidant enzyme. It plays a key role in reducing H_2_O_2_ and lipid peroxides (LOOH) by utilizing GSH (Weaver and Skouta, 2022). The rs713041 SNP within the *GPX4* gene introduces a C-T substitution, specifically located in the 3′untranslated region (3′UTR) of the mRNA. This region plays a crucial role in selenoprotein synthesis facilitating the incorporation of Secys. A genetic variation in this region has the potential to influence GPx4 activity, particularly under conditions of low selenium intake, rendering individuals more susceptible to oxidative stress-related diseases (Barbosa et al., 2022). The rs713041 polymorphism in *GPX4* gene presents three distinct genotypes, CC (Homozygous wild), CT (Heterozygous), TT (Homozygous mutant). In this polymorphism, the C allele appears to confer a protective role against oxidative damage, particularly when selenium levels are sufficient. It also contributes to maintaining GPx4 concentrations in lymphocytes, particularly for individuals with the CC genotype, compared to those with the TT genotype in situations of inadequate selenium intake (Barbosa et al., 2022). The substitution of C allele with T allele has been linked to conditions such as obesity, endometriosis, thyroid diseases, Alzheimer’s disease, depression, multiple sclerosis, and various possibly owing to its implication in oxidative stress (Barbosa et al., 2022; Weaver and Skouta, 2022).

### Glutathione system

The glutathione system, anchored by glutathione (GSH), stands as a critical defense mechanism against oxidative stress. GSH, a tripeptide composed of L-glutamate, L-cysteine, and glycine, plays a pivotal role in maintaining cellular redox balance, essential for overall health (Deponte, 2022) (Supplementary Figure S2). Its synthesis is orchestrated by two key enzymes, γ-Glutamyl cysteine synthase and glutathione synthetase (GSS), fueled by ATP hydrolysis within the cytosol (Lu, 2013; Deponte, 2022). Additionally, glutamate-cysteine ligase (GLUL) catalyzes the formation of gamma-glutamylcysteine, a precursor to GSH, in the initial stage of GSH synthesis. GSH functions as a crucial substrate for enzymes like GPx, which scavenge peroxides to protect cells from oxidative damage. Glutathione reductase (GR) aids in GSH regeneration by converting oxidized glutathione (GSSG) back to its active form, thereby maintaining an optimal cellular pool of GSH for antioxidant defense and redox homeostasis (Lu, 2013) (Supplementary Figure S2). Furthermore, glutathione S-transferases (GSTs), including GSTM, GSTP, and GSTA, among others, contribute to detoxification processes within cells. These enzymes facilitate the conjugation of GSH with electrophilic compounds, enhancing their solubility and facilitating their removal from the cell. By neutralizing and eliminating harmful substances, GSTs play a crucial role in protecting cells from oxidative damage and maintaining overall cellular health (Nissar et al., 2017; Sarıkaya and Doğan, 2020) (Supplementary Figure S2). Together, these elements form the robust, glutathione defense network crucial for cellular health and resilience against oxidative insults. Polymorphisms in these enzymes have been associated with various diseases.

The *GSS* gene encodes the glutathione synthetase enzyme (GSS), a critical player in the synthesis of GSH (Kalamkar et al., 2023). GSS catalyzes the final step in GSH biosynthesis, using ATP to ligate γ-glutamylcysteine with glycine. This is the final step in the synthesis of GSH (Lu, 2013) Polymorphisms rs121909307 in the *GSS* gene can impact the activity of the GSS enzyme, influencing the production of GSH and, consequently, the cellular response to oxidative stress. Individuals with CC genotype exhibit optimum GSS activity, resulting in a lower risk of oxidative stress. In contrast, those with CT or TT genotypes experience reduced GSS activity, leading to decreased GSH production and a higher susceptibility to oxidative stress. The polymorphic variations in the *GSS* gene directly correlate with the enzyme’s function, influencing the cellular antioxidant capacity and the ability to combat oxidative stress (Kalamkar et al., 2023). Individuals carrying the CT or TT genotypes may face an increased risk of conditions where oxidative stress plays a pivotal role, such as neurodegenerative disorders, cardiovascular diseases, or certain types of cancers (Kalamkar et al., 2023).

The *GLUL* gene encodes the enzyme glutamate ammonia ligase, also known as glutamine synthetase. This enzyme is vital for maintaining cellular levels of glutamine, an amino acid with various functions, including antioxidant properties (Halama and Suhre, 2022). Glutamine is a precursor for GSH synthesis, crucial for controlling cellular redox status, highlighting the importance of the *GLUL* gene (Halama and Suhre, 2022). Polymorphisms in the *GLUL* gene, particularly, rs10911021 contribute to variations in oxidative stress susceptibility. Homozygous wild individuals (TT) have sufficient levels of glutamine synthetase and glutathione experience lower oxidative stress. Heterozygous individuals (TC) with decreased levels of glutamine synthetase enzyme and glutathione may face an increased risk of oxidative stress. Homozygous mutant individuals (CC) with reduced levels of glutamine synthetase enzyme and glutathione exhibit heightened susceptibility to oxidative stress (Tonin et al., 2024).

The *GSR* gene produces the glutathione-disulfide reductase protein, also known as the glutathione reductase (GR) enzyme. This enzyme plays a crucial role in maintaining the reduced form of GSH. This action mediated by GR is integral for replenishing the pool of GSH (Vyas et al., 2022). Mutations in the *GSR* gene can cause hereditary glutathione reductase deficiency, affecting cellular redox potential and increasing oxidative stress levels, especially in red blood cells. This deficiency is linked to conditions such as hereditary hemolytic anemia (Vyas et al., 2022). In the polymorphism, rs8190955 in the *GSR* gene, individuals with the C allele have optimum levels of GR while individuals with T allele are associated with a GR deficiency. As a result, homozygous wild individuals with the CC genotype have appropriate antioxidant function and lower levels of oxidative stress in red blood cells. On the other hand, heterozygous individuals and homozygous mutant individuals with the CT and TT genotypes, respectively, have impaired cellular redox potential and increased oxidative stress levels in red blood cells, owing to the GR deficiency. This deficiency is associated with hereditary hemolytic anemia (Vyas et al., 2022).

Glutathione transferases (GST) form a critical enzyme family in cellular detoxification and defense against oxidative stress. They facilitate the conjugation of GSH with electrophilic compounds, aiding in the elimination of harmful substances. These enzymes are categorized into cytosolic, mitochondrial, and microsomal members and are classified into multiple classes including Alpha (A), Mu (M), and Pi (P), each with distinct subtypes. GSTs are expressed predominantly in the liver and are involved in metabolizing various compounds. Their primary function lies in rendering substances more water-soluble for excretion (Hayes and McLellan, 1999). GSTs are crucial for maintaining cellular homeostasis and preventing the accumulation of toxic compounds, highlighting their role in cellular health maintenance. Genetic polymorphisms in GST genes can alter enzyme activity and may exhibit altered detoxification capacities and altered redox state, affecting susceptibility to diseases such as cancer, neurodegenerative disorders, and cardiovascular diseases (Grussy et al., 2023).

The *GSTM1* gene produces an enzyme called glutathione S-transferase Mu 1 (GSMT1). The enzyme is involved in detoxifying toxic compounds by catalyzing the conjugation of GSH with a variety of electrophilic substrates, which makes the compounds more water-soluble and facilitating their elimination from the body. Found in cellular compartments such as mitochondria, lysosomes, and nuclei, this GSTM1 safeguards organelles, especially the mitochondria from oxidative stress. It achieves this by preventing cardiolipin peroxidation and cytochrome c release, making it a key regulator in fighting ROS (Mortazavi et al., 2020). Polymorphisms in the *GSTM1* gene contribute to variations in oxidative stress susceptibility. The rs366631 polymorphism is characterized by the T>C change. Individuals with the TT genotype exhibit normal GSTM1 activity and have normal ROS scavenging abilities. On the other hand, individuals with the CT and CC genotypes display reduced GSTM1 activity, making them prone to oxidative stress due to the gene’s diminished ability to scavenge oxidant species (Mortazavi et al., 2020; Dai et al., 2021).

Similarly, the *GSTM5* gene is part of the GST family and encodes the enzyme, glutathione S-transferase 5 (GSTM5) found in cellular compartments, such as the mitochondria. The enzyme is crucial for protecting cell organelles from oxidative stress. For rs3754446 polymorphism in GSTM5 individuals with TT genotypes exhibit normal GSTM5 activity, associated with normal mitochondrial function and a lower risk of oxidative stress. In contrast, individuals with GT and GG genotypes have altered GSTM5 activity and experience heightened oxidative stress due to ROS accumulation in the mitochondria (Butrym et al., 2021).

Glutathione S-transferase P1 (GSTP1) encoded by the *GSTP1* gene, is a crucial enzyme found throughout various cellular compartments such as the cytoplasm, mitochondria, lysosomes, and nucleus. Its mitochondrial form plays a vital role in protecting organelles from oxidative stress by inhibiting cardiolipin peroxidation and preventing cytochrome c release (Santric et al., 2020). Polymorphisms within the *GSTP1* gene, such as rs1138272 contribute to variations in oxidative stress susceptibility. Individuals with the AA genotype exhibit normal gene activity, leading to appropriate antioxidant activity and a lower risk of oxidative stress. In contrast, those with the AG genotype show partially abnormal gene activity, resulting in decreased antioxidant activity and an elevated risk of oxidative stress. Homozygous GG individuals experience decreased gene activity, reduced antioxidant capacity, and a higher risk of oxidative stress. These polymorphic variations directly impact the enzyme’s activity, influencing the cellular response to oxidative stress conditions (Santric et al., 2020).

### Thioredoxin system

The thioredoxin system is crucial for regulating redox processes. It consists of thioredoxin (Trx) and its partner, thioredoxin reductase (TR or TrxR), which uses NADPH to reduce Trx (Lee et al., 2013) (Supplementary Figure S3). Trx acts as an antioxidant by transferring electrons and protons, converting disulfides into dithiols (Lee et al., 2013). Trx maintains its active state mainly through the action of TR. It can also be reactivated by glutaredoxin (Grx) within the glutathione system. Trx serves as an antioxidant by directly quenching singlet oxygen (^1^O_2_) and hydroxyl radicals (^⋅^OH) or indirectly by reducing oxidized proteins. A significant target of Trx is peroxiredoxin (Prx), which directly reduces peroxides including H_2_O_2_ and various alkyl hydroperoxides. After Prx reduces its target, Trx recycles the oxidized form of Prx back to its reduced state (Hanschmann et al., 2013). Overall, the thioredoxin system collaborates with the glutathione system to maintain the organism’s redox balance and protect against oxidative stress.

The *TXN2* gene encodes thioredoxin-2, that reduces Prx dimers that are formed upon reaction with H_2_O_2_, thereby keeping Prx in their reduced and active state (Supplementary Figure S3). TXN2 is essential in particular for the efficient cycling of PRDX3, which indicates its importance in the body’s antioxidant defences (Lee et al., 2013). Genetic variations within the *TXN2* gene, particularly the rs35045487 polymorphism is known to be crucial in modulating of oxidative stress. This polymorphism, located in the proximal promoter region, involves an insertion/deletion impacting the transcriptional activity (Hanschmann et al., 2013). Alleles A2 (GA insertion), A4 (G insertion), and A5 (GGGA insertion) display decreased transcriptional activity, attributed to additional SP1 binding sites. This suggests a potential association with heightened oxidative stress, indicating that individuals carrying these alleles may be predisposed to an imbalance in redox homeostasis (Wen et al., 2009). Similarly, the rs4485648 polymorphism in intron one of the *TXN2* gene is known to modulate oxidative stress-risk. The variant, ‘TT’ and ‘CT’ alleles of this polymorphism may have altered TXN2 expression which may compromise its functionality leading to oxidative stress. On the other hand, the ‘CC’ genotypes have appropriate gene expression associated with optimum antioxidant function. A study showed that the TT and CT genotypes were associated with the increased risk of diabetic retinopathy which could be mediated by elevated oxidative stress (Ramus et al., 2016).

### Heme oxygenase - 1

Heme oxygenase (HO) plays a crucial role in regulating oxidative stress by maintaining heme homeostasis. There are three isoforms of heme oxygenase: HO-1, HO-2, and HO-3. Among these, HO-1 is upregulated in response to various stress stimuli, including oxidative stress. Its activation is a protective response against oxidative stress, as it helps to degrade heme, a pro-oxidant molecule, and generates products like biliverdin, which possess antioxidant properties HO-1 expression is regulated by the transcription factor, Nrf2, which activates antioxidant response elements (AREs) in the promoter region of the *HMOX1* gene, encoding HO-1 (Chiang et al., 2021; Ryter, 2022). One notable polymorphism in the *HMOX1* gene is rs2071746, where the A>T change is linked to various oxidative stress-related diseases like sickle cell anemia, ischemic heart disease, hypertension, and rheumatoid arthritis. Particularly in sickle cell anemia, the rs2071746TT genotype in the *HMOX1* gene’s promoter is associated with elevated fetal hemoglobin (Hb F) levels. The T allele of rs2071746 is linked to reduced gene expression, potentially leading to higher free heme concentration and stress-induced erythropoiesis, consequently increasing Hb F levels. This association may contribute to the heightened oxidative stress observed in sickle cell anemia. (Pandey et al., 2023). Table 2 summarizes the mechanisms by which genetic polymorphisms influence antioxidant genes.

**TABLE 2:** Mechanisms of genetic polymorphisms affecting antioxidant genes.

Antioxidant enzyme	Gene name	rsID	Function of mutation	Refe rences
Superoxide dismutase	SOD1	rs2234694	Reduced SOD1 enzyme activity hampers the conversion of O_2_^⋅-^ to H_2_O_2_ and O_2_, dysregulating redox balance	Ben Anes et al. (2019), Eleutherio et al. (2021)
		rs36232792	Decreased promoter activity results in lower SOD1 enzyme synthesis, impairing its ability to neutralize O_2_^⋅-^ radicals	Gallegos-Arreola et al. (2020)
	SOD2	rs4880	Accelerated degradation of SOD2 mRNA lowers SOD2 activity, potentially leading to increased oxidative stress	Griess et al. (2017)
	SOD3	rs1799895	Impaired SOD3 (ECSOD) binding to the ECM reduces tissue SOD3 levels, leading to decreased protection against oxidative damage	Ighodaro and Akinloye (2018)
Catalase	CAT	rs1001179, rs769217	Lower catalase activity and expression affects the enzyme’s ability to neutralize intracellular H_2_O_2_	Toppo et al. (2008), Shaparov et al. (2021)
		rs769214	Increased catalase transcriptional activity resulting in improved antioxidant function	Flohé and Brigelius-Flohé (2011), Mohammedi et al. (2016)
Glutathione Peroxidase	GPX1	rs1050450	Affected catalytic enzyme activity, substrate affinity, and structural stability which may lower GPx1’s ability to combat oxidative stress	Deponte (2022)
	GPX3	rs3805435	Reduced GPx3 enzyme levels resulting in poor antioxidant defenses and heightened oxidative stress	Nissar et al. (2017)
	GPX4	rs713041	Affected selenoprotein synthesis impacting GPx4 activity and potentially increasing susceptibility to oxidative stress	Tonin et al. (2024)
Glutathione synthetase	GSS	rs121909307	Altered GSS enzyme activity, influencing the production of GSH and consequently, affecting cellular response to oxidative stress	Hanschmann et al. (2013)
Glutamate ammonia ligase	GLUL	rs10911021	Decreased levels of the glutamine synthetase enzyme and GSH, resulting in the increased risk of oxidative stress	Chiang et al. (2021)
Glutathione reductase	GSR	rs8190955	Lower GR levels leading to impaired cellular redox potential caused by the affected antioxidant pool; results in increased oxidative stress levels, especially in red blood cells	Chiang et al. (2021)
Glutathione transferases	GSTM1	rs366631	Reduced GSTM1 activity affects the conjugation of GSH to toxic products leading to their poor elimination and compromising GSTM1’s ROS scavenging abilities	
	GSTM5	rs3754446	Reduced mitochondrial GSTM5 activity affects GSH’s conjugation to toxic products resulting in their poor elimination, thereby compromising GSTM5’s ability to quench ROS in the mitochondria	
	GSTP	rs1138272	Abnormal GSTP1 activity decreases cellular antioxidant capacity	
Thioredoxin	TXN2	rs35045487, rs4485648	Decreased transcriptional activity leads to altered TXN2 gene expression reducing its antioxidant function	
Heme-oxygenase	HO-1	rs2071746	Reduced gene expression raises free heme concentration, promoting stress-induced erythropoiesis and increasing Hb F levels, thereby leading to oxidative stress, especially in sickle cell anemia	

Abbreviations: SOD1, Superoxide Dismutase 1; O_2_^⋅-^, Superoxide; H_2_O_2_, hydrogen peroxide; O_2_, Oxygen; SOD2, Superoxide Dismutase 2; SOD3, Superoxide Dismutase 3; ECSOD, Extracellular superoxide dismutase; ECM, Extracellular matrix; GPx1, Glutathione peroxidase 1; GPx3, Glutathione peroxidase 3; GPx4, Glutathione peroxidase 4; GSS, Glutathione synthetase; GSH, Glutathione; GR, Glutathione reductase; GSTM1, Glutathione S-transferase Mu 1; ROS, Reactive oxygen species; GSTM5, Glutathione S-transferase 5; GSTP1, Glutathione S-transferase P1; TXN2, Thioredoxin-2; Hb F, Fetal hemoglobin.

## Conclusion

The review has explored the intricate relationship between genetic predispositions and oxidative stress which could be associated with the pathogenesis of various conditions. Through the assessment of single nucleotide polymorphisms (SNPs) relevant to oxidative stress, we have highlighted the significant impact of genetic variations in the prooxidant genes, XDH, CYBA, CYP1A1, PTGS2, NOS, MAO and the antioxidant genes, SOD, CAT, GPX, GSS, GLUL, GSR, GSTM1, GSTM5, GSTP1, TXN, and HMOX1 on oxidative stress susceptibility. These polymorphic variations can influence the expression and activity of the encoded proteins, thereby disrupting the delicate redox balance in the body.

Genetic assessment aids in understanding enzyme and pathway variations associated with oxidative stress, offering insights into individuals’ innate potential to produce and combat oxidant species. It allows for early detection of those at higher risk of oxidative stress-related conditions, enabling timely mitigation strategies. Integration of genetic insights into treatment facilitates personalized medicine tailored to one’s genetic profile. Additionally, assessment of biological markers represents actual oxidative stress levels in the body, crucial for managing oxidative stress effectively.

Furthermore, ongoing clinical trials explore various antioxidant approaches, including boosting glutathione levels using precursors, enhancing the synthesis of antioxidant enzymes, particularly via NRF2 pathway activation, NOX inhibition, and mimics such as SOD, GPX and catalase, to mitigate oxidative stress and associated pathologies. Further research is needed to optimize these interventions and understand their efficacy in managing oxidative stress-related conditions.

In conclusion, this review sheds light on the current understanding of genetic determinants of prooxidants and antioxidants offering a comprehensive perspective on how variations in these genes can modulate the risk of oxidative stress. Moving forward, further research is warranted to elucidate the precise molecular mechanisms underlying these genetic associations and to develop targeted interventions for mitigating the adverse effects of oxidative stress on health.

## Abbreviations

CAT, Catalase; Fe^2+^, Ferrous iron; GPx, Glutathione peroxidase; GSH, Reduced glutathione; GST, Glutathione S-transferase; NO, Nitric oxide; NOS, Nitric oxide synthase; O_2_^2^ˉ, Superoxide; ONOO^−^, Peroxynitrite; ROS, Reactive oxygen species; SOD, Superoxide dismutase; H_2_O_2_, Hydrogen peroxide; ⋅OH, Hydroxyl radicals; TPx, Thioredoxin peroxidase.
