# Supplementary material for: Inside the genome: understanding genetic influences on oxidative stress
Source: Front Genet. 2024 Jun 25;15:1397352. doi: 10.3389/fgene.2024.1397352 (PMC11231378; doi:10.3389/fgene.2024.1397352)
Supplement: Supplementary file 1 [file Presentation1.PDF]

Inside the Genome: Understanding Genetic Influences on Oxidative Stress

Hari Krishnan Krishnamurthy[1]*, Imbaasree Rajavelu[2], Michelle Pereira[2], Vasanth Jayaraman[1], Karthik Krishna[1], Tianhao Wang[1], Kang Bei[1], John J. Rajasekaran[1]

1 Vibrant Sciences LLC., San Carlos, CA, United States of America,

2 Vibrant America LLC., San Carlos, CA, United States of America

Acknowledgement: We acknowledge Vibrant America LLC for supporting this research.

Correspondence to: *Hari Krishnan Krishnamurthy
Contact no. 5094325707 Email address: hari@vibrantsci.com

Author information

Author	Email address	Highest academic degree	Affiliations
Hari Krishnan Krishnamurthy	hari@vibrantsci.com	Master's degree of science	Vibrant Sciences LLC., San Carlos, CA, USA
Imbaasree Rajavelu	imbaasree.r@vitasoft-tech.com	Master's degree of science	Vibrant America LLC., San Carlos, CA, USA
Michelle Pereira	michelle.p@vitasoft-tech.com	Master's degree of science	Vibrant America LLC., San Carlos, CA, USA
Vasanth Jayaraman	vasanth.jayaraman@vibrantsci.com	Master's degree of science	Vibrant Sciences LLC., San Carlos, CA, USA
Karthik Krishna	karthik@vibrantsci.com	Master's degree of science	Vibrant Sciences LLC., San Carlos, CA, USA
Tianhao Wang	tianhao.wang@vibrantsci.com	Master's degree of science	Vibrant Sciences LLC., San

			Carlos, CA, USA
Kang Bei	kang@vibrantsci.com	Master's degree of science	Vibrant Sciences LLC., San Carlos, CA, USA
John J. Rajasekaran	jjrajasekaran@vibrantsci.com	Doctor of Philosophy	Vibrant Sciences LLC., San Carlos, CA, USA

SUPPLEMENTARY MATERIAL

FIGURES

Figure 1

THE NOS PATHWAY IN OXIDATIVE STRESS

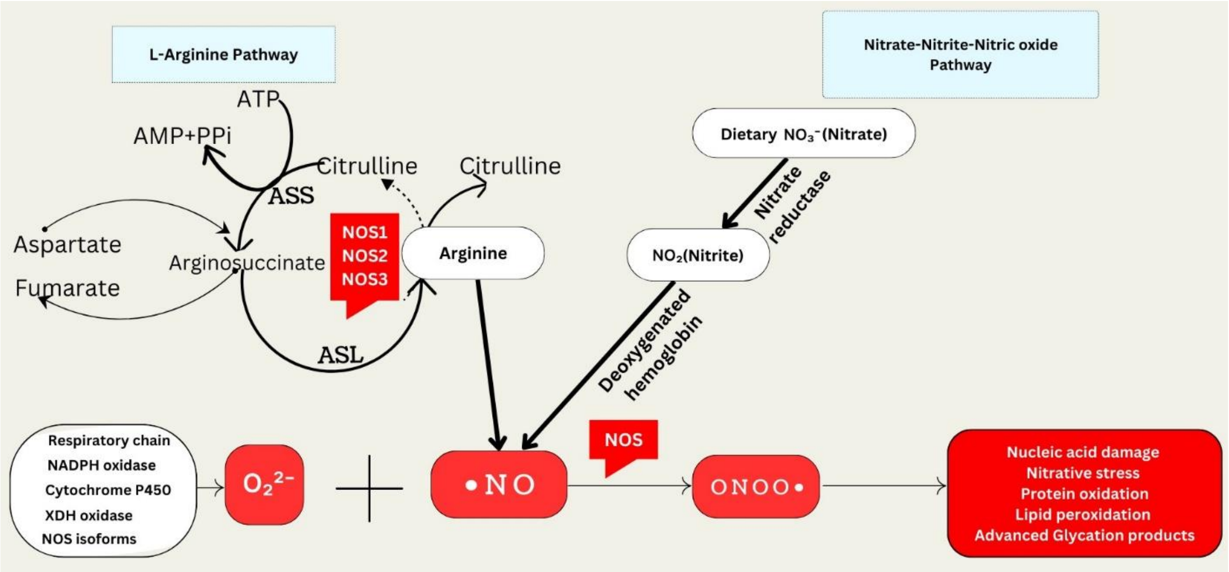

Figure 1. The NOS Pathway in Oxidative Stress

Abbreviations
NOS - Nitric oxide synthase
$^{\bullet}$NO - Nitric oxide
O_2^{2-} - Superoxide
$ONOO^{-}$ - Peroxynitrite

Figure 1. The NOS Pathway in Oxidative Stress. The figure illustrates the multifaceted pathway involving NOS in oxidative stress conditions. Under normal circumstances, NOS catalyzes the conversion of L-arginine to L-citrulline, producing $^{\bullet}$NO, a crucial signaling molecule. Additionally, dietary intake of nitrate and nitrite can contribute to $^{\bullet}$NO production through the nitrate-nitrite-NO pathway. However, in the presence of O_2^{2-}, generated during oxidative stress, $^{\bullet}$NO reacts to form $ONOO^{-}$, a potent oxidant. This reaction leads to the synthesis of peroxynitrite, exacerbating oxidative stress and its detrimental effects on cellular components. The intricate interplay between NOS, O_2^{2-}, and NO underscores the significance of oxidative stress in various pathological conditions.

Figure 2

GLUTATHIONE PATHWAY IN OXIDATIVE STRESS

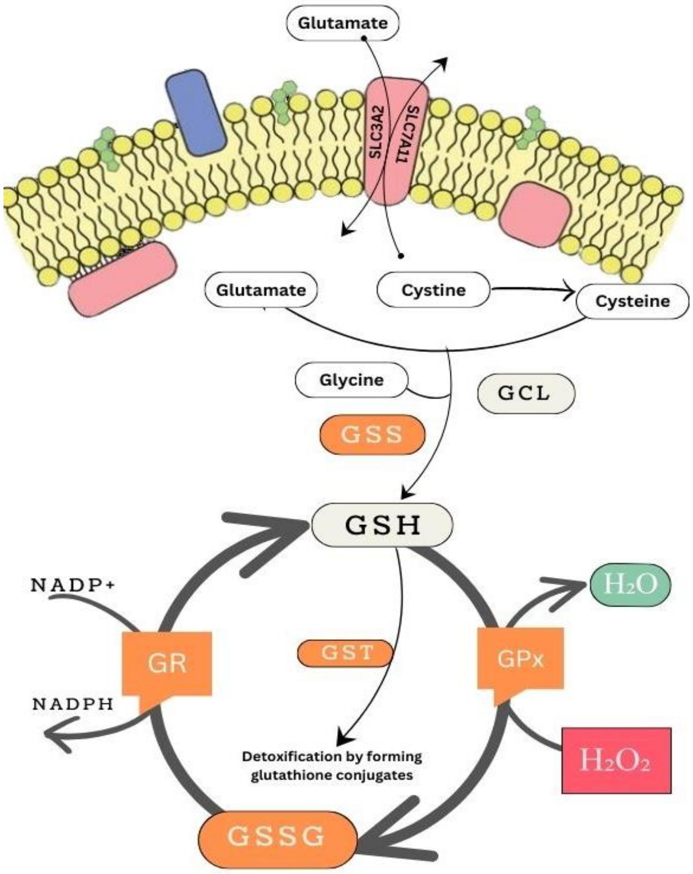

Figure 2: Glutathione Pathway in Oxidative Stress

Abbreviations

GSH – Glutathione
GCL - Glutamate-cysteine ligase
GSS - Glutathione synthetase
H_2O_2 - Hydrogen peroxide
ROS - Reactive oxygen species
GSSG - Oxidized glutathione
GR – Glutathione Reductase

Figure 2: Glutathione Pathway in Oxidative Stress. This schematic illustrates the GSH pathway in oxidative stress, showcasing the synthesis of GSH and its role in the glutathione cycle. Glutamate enters the cell membrane from the extracellular space via specific transporters. Inside the cell, glutamate combines with cysteine and glycine, facilitated by the enzymes GCL and GSS, to form GSH. GSH is a critical antioxidant involved in cellular defense against oxidative stress. In the glutathione cycle, GSH reacts with ROS such as H_2O_2, catalyzed by the enzyme glutathione peroxidase GPx, to form GSSG and water. GSSG is then converted back to GSH through the action of GR, utilizing NADPH as a cofactor. This regeneration of GSH enables its continued function in scavenging ROS and maintaining redox balance within the cell. Overall, the glutathione pathway plays a crucial role in mitigating oxidative stress by synthesizing GSH, which acts as a potent antioxidant, and by recycling GSSG back to GSH, thus efficiently neutralizing harmful ROS like H_2O_2 to harmless water.

Figure 3

THIOREDOXIN PATHWAY IN OXIDATIVE STRESS REGULATION

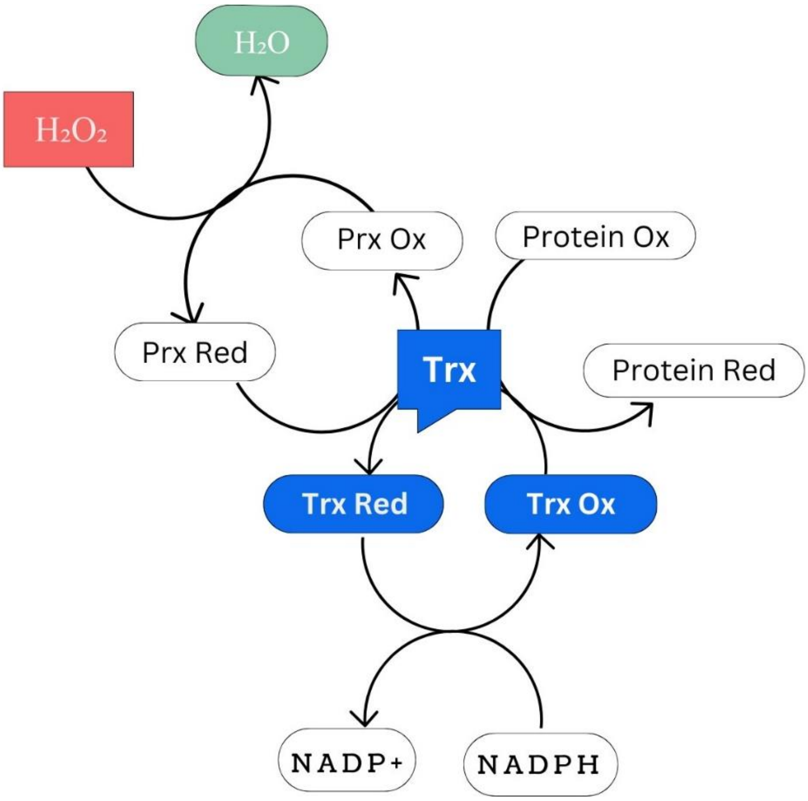

Figure 3: Thioredoxin Pathway in Oxidative Stress Regulation.

Abbreviations
Trx – Thioredoxin
ROS - Reactive oxygen species
Trx-S2 - Oxidized state
Trx-SH - Reduced form
Prx Ox - Oxidized peroxiredoxins
Prx Red – Reduced peroxiredoxins
H_2O_2 - Hydrogen peroxide
H_2O - Water

Figure 3: Thioredoxin Pathway in Oxidative Stress Regulation. The diagram illustrates the Trx pathway, a crucial cellular defense mechanism against oxidative stress. In response to elevated ROS, Trx undergoes reduction by NADPH-dependent thioredoxin reductase, transforming from its Trx-S2 to its reduced form Trx-SH. The reduced Trx-SH, in turn, functions as a potent electron donor for the reduction of Prx Ox to Prx Red, essential peroxidases involved in ROS detoxification. Concurrently, Trx activates various redox-sensitive proteins by reducing their disulfide bonds, restoring their functional state. Notably, this pathway plays a pivotal role in maintaining cellular homeostasis by facilitating the conversion of H_2O_2 to H_2O, thereby mitigating oxidative damage and preserving cellular integrity.
